# Modeling of Osmotic Dehydration of Apples in Sugar Alcohols and Dihydroxyacetone (DHA) Solutions

**DOI:** 10.3390/foods8010020

**Published:** 2019-01-09

**Authors:** Joanna Cichowska, Adam Figiel, Lidia Stasiak-Różańska, Dorota Witrowa-Rajchert

**Affiliations:** 1 Department of Food Engineering and Process Management, Faculty of Food Sciences, Warsaw University of Life Sciences WULS-SGGW, 159c Nowoursynowska St., 02-776 Warsaw, Poland; dorota_witrowa_rajchert@sggw.pl; 2 Institute of Agricultural Engineering, Wrocław University of Environmental and Life Sciences, 37a Chełmońskiego St., 51-630 Wrocław, Poland; adam.figiel@upwr.edu.pl; 3 Department of Biotechnology, Microbiology and Food Evaluation, Faculty of Food Sciences, Warsaw University of Life Sciences WULS-SGGW, 159c Nowoursynowska St., 02-776 Warsaw, Poland; lidia_stasiak_rozanska@sggw.pl

**Keywords:** osmotic dehydration, water loss, sugar alcohols, polyols, dihydroxyacetone, modeling

## Abstract

The purpose of this paper is twofold: on the one hand, we verify effectiveness of alternatives solutes to sucrose solution as osmotic agents, while on the other hand we intend to analyze modeling transfer parameters, using different models. There has also been proposed a new mass transfer parameter—true water loss, which includes actual solid gain during the process. Additional consideration of a new ratio (Cichowska et al. Ratio) can be useful for better interpretation of osmotic dehydration (OD) in terms of practical applications. Apples v. Elise were dipped into 30% concentrated solutions of erythritol, xylitol, maltitol, and dihydroxyacetone (DHA) to remove some water from the tissue. To evaluate the efficiency of these solutes, 50% concentrated sucrose solution was used as a control. All of the tested osmotic agent, except maltitol, were effective in the process as evidenced by high values in the true water loss parameter. Solutions of erythritol and xylitol in 30% concentrate could be an alternative to sucrose in the process of osmotic dehydration. Peleg’s, Kelvin–Voigt, and Burgers models could fit well with the experimental data. modeling of mass transfer parameters, using Peleg’s model can be satisfactorily supplemented by Kelvin–Voigt and Burgers model for better prediction of OD within the particular periods of the process.

## 1. Introduction

The osmotic agent most commonly used in the osmotic dehydration (OD) of fruits is sucrose [1]. There are a lot of papers concerning using sucrose as an osmotic agent in the process of osmotic dehydration of apple, kiwifruit, cranberry, and pineapple [2,3,4,5]. Due to the increasing interest in designing new, low calorie, and minimally processed foods, the replacement of sugar in traditional candied products and study of osmotic dehydration kinetics with the use of alternative carbohydrates has become a growing challenge [6]. Nowadays, it is important to find new alternative to sucrose solutions, which can be used in this process. Recently this topic has become more popular. Sugar alcohols were used as solutes in the OD process [7,8,9,10]. Sugar alcohols (the polyols or polyhydric alcohols) are low digestible carbohydrates, which are obtained by substituting an aldehyde group with a hydroxyl one. Polyols have lower caloric value (from 0.2 to 2.7 kcal/g, compared with sucrose 4 kcal/g). Moreover, consumption of the products containing sugar alcohols do not induce an increase in blood glucose or insulin secretion, and thus, such products are recommended for the people with diabetes [11].

1,2,3,4-Butanetetrol, which is a chemical name of erythritol, can be naturally found in a wide variety of fruits (melons, peaches, grapes), vegetables, and fermented foods such as wine, beer, sake, and soy sauce [12]. Erythritol is very stable and does not decompose in either acid or alkaline environments. It does not contain a reducing end group, thereby it does not take part in Maillard-type browning reactions. Using erythritol, it is possible to obtain sugar-free, low-calorie, non-cariogenic fondants, candies, chocolates, ice creams, chewing gums, etc. [13]. Based on clinical studies, it was stated that erythritol is well (60–90%) and rapidly absorbed in the small intestine and then excreted intact in urine within 24 h. Gastrointestinal effects were recorded when high doses were consumed, up to 1000 mg/kg body [14]. While all other polyols have the distinct disadvantage of causing digestive distress, erythritol is well-tolerated. The key factor in erythritol’s high level of digestive tolerance is its small molecular size [12].

Xylitol is a 5-carbon polyol, which is produced from d-xylose. It can be found in nature in many fruits (yellow plums, strawberries, raspberries, bilberries) and vegetables (cauliflower, lettuce, spinach, aubergine), oats, and mushrooms, and is produced in small quantities in the human organism. As it contains no reducing groups, it will not take part in Maillard reactions. Xylitol is approximately absorbed in 50% in the small intestine whereas its fermentation, which takes place in the large bowel, ranges from 50 to 75%. Human tolerance of xylitol amounts to 100 g per day and following the ingestion of xylitol, the blood glucose and serum insulin responses are significantly lower than those following glucose or sucrose ingestion. Xylitol is an ideal sweetener for developing high-quality sugar-free products, as its sweetness and cooling effect creates excellent tasting sugar-free confectionery with added health benefits. Commercially, the examples of xylitol-containing products exist for most confectionery types, including chewy candy, hard-boiled candy, gum Arabic pastilles, gelatin jellies, pectin jellies, starch jellies, toffees, caramels fondant, fudge, cast lozenges, compressed tablets, and mini-mints [11,15].

Maltitol (4-O-α-*d*-glucopyranosyl-*d*-glucitol), is a disaccharide polyol that consists of glucose and sorbitol in equal parts. Maltitol is characterized by a pleasant sweet taste. However, due to its slow absorption, the insulin response associated with its ingestion is significantly reduced. It does not undergo carmelization or browning processes, and its cooling effect is negligible when compared with other polyols. It can also be used as a fat substitute, since it gives a creamy texture to food. Due to its low hygroscopicity and stability in high temperatures, it is used in many baked products as well as in a variety of reduced calorie, reduced fat, and sugar-free foods. Although its ADI (acceptable daily intake) was not specified, it reveals laxative effects when consumed in amounts exceeding 25–30 g/kg body weight per day [11,16].

Worldwide production increment of biodiesel involves the need for management of by-products of the process. With the production of 10 kg of biodiesel from rapeseed oil, 1 kg of glycerol becomes available [17]. With the growing production of biodiesel in the coming years, managing the crude glycerol production will become an increasingly difficult task. Pure glycerol is used as a raw material and can be converted into various value added products in the personal care, cosmetics, pharmaceutical and food industries [18]. It is possible to gain valuable compound—dihydroxyacetone (DHA) by incomplete oxidation of glycerol with acetic bacteria. DHA is ketotriose with reducing properties, non-toxic sugar in the form of white powder with a sweet taste. It is used mainly in the food industry (a sweetener, a dietary supplement, emulsifier, plasticizer), cosmetics (the active ingredient in self-tanning creams) and medicine (treatment of vitiligo disease, a component of biomaterials stopped bleeding) [19]. There have not been any studies so far regarding the use of dihydroxyacetone as a solute in the process of OD. Due to the almost 4-times lower molecular weight (90.08 g/mol), compared to sucrose (342.30 g/mol), there is a potential to create a greater difference of osmotic pressure in the system (which is the driving force of the OD process).

Mathematical modeling may be performed to describe the OD mass transfer kinetics, and, consequently, to study the effects of process variables on the process or predict selected responses during food processing [20]. Farzaneh et al. [21] used a model relying on fuzzy logic to predict optimal conditions of 15 properties of bean, depending on the moisture content. The models may be classified as empirical and semi-empirical, phenomenological, and mechanistic [22]. It is important to choose a correct model to present the results. This research aims to evaluate modeling mass transfer kinetics of osmotic dehydration using different models, as well as verify effectiveness alternatives solutes to sucrose solution as osmotic agents. There has also been proposed a new mass transfer parameter—true water loss, which includes actual solid gain during the process.

## 2. Materials and Methods

### 2.1. Sample Preparation

Fresh apples of the Elise variety were collected from the Experimental Fields (Orchards) of the Faculty of Horticulture and Landscape Architecture (Warsaw University of Life Sciences). The fruits were stored at 4 ± 1 °C and relative humidity of 85–90% in a refrigerator until use. Before the experiment, the apples were washed, pitted and cut into 5 mm thick slices and then each slice into 4 pieces.

### 2.2. Pre-Treatment Procedure

The slices were dehydrated by osmotic dehydration (OD) in a water bath (Water Bath Shaker Type 357 ELPAN, Poland) with continuous shaking (1 Hz amplitude). Apple samples were dipped into 30% concentrated syrups. Osmotic solutions were prepared with selected substances of sugar alcohols: erythritol (F8030, Brenntag), xylitol (Brenntag), maltitol (Brenntag), as well as dihydroxyacetone (DHA) (Merck, Germany) and distilled water. To compare the process kinetics, 50% sucrose solution was used in the experiment. Osmotic dehydration was carried out in the range from 30 to 180 min (the optimal process conditions—time and concentration—were selected for testing based on preliminary experiments in a previous work published by Cichowska et al. [10]) at a temperature of 40 °C (atmospheric pressure) in ratio of 1:4 (fruit: solution) [23,24]. The mass of a single sample was 20 g ± 2 g. Afterwards, the samples were removed from the osmotic solution and blotted with absorbent paper to remove osmotic liquid from their surface. Two technological repetitions were performed for each treatment.

### 2.3. Mathematical Modeling

The kinetic parameters have been calculated in all the cases: water content (*WC*), solids gain (*SG*), water loss (*WL*), at the different time (*τ*) according to equations [10]:

(1) $$WC=\frac{1-s_{\tau }}{s_{o}}$$

(2) $$SG=\frac{s_{\tau }\times m_{\tau }-s_{o}\times m_{o}}{s_{o}\times m_{o}}$$

(3) $$WL=\frac{\left(1-s_{o}\right)\times m_{o}-\left(1-s_{\tau }\right)\times m_{\tau }}{s_{o}\times m_{o}}$$

In this paper, we propose a new mass transfer parameter, which is a true water loss, *WL_T_*. This value informs about the real amount of water loss (no per 1 g of initial dry matter, but includes solid gain during the process):

(4) $$WL_{T}=\frac{WL_{\tau }}{1+SG_{\tau }}$$

The ratio of true water loss to water loss we named Cichowska’s et al. Ratio (CR):

(5) $$CR=\frac{WL_{T}}{WL}$$

In this work *SG*, *WL* and *WL_T_* data (Equations (2)–(4)) were fitted using three models.

The Peleg’s model [25]:

(6) $$Y=Y_{o}\pm \frac{\tau }{\left(k_{1}+k_{2}\tau \right)}$$

where parameters *k*_1_ and *k*_2_ are the known Peleg’s constants [26]; the Kelvin–Voigt model [27]:

(7) $$Y=A*\left(1-exp^{\frac{-\tau }{B}}\right)$$

which could be also presented in another form [6]:

(8) $$Y=A*\left(1-exp^{-K\tau }\right)$$

and the Burgers model [27]:

(9) $$Y=A*\left(1-exp^{\frac{-\tau }{B}}\right)+C*\tau$$

were employed to fit the experimental results.

Parameter CR (Equation (9)) was modeled using Peleg’s Equation (6), when Y_0_ was equal 1.

Fitting of the mathematical functions (Peleg, Kelvin–Voigt, and Burgers) to the experimental points was done using Table Curve 2D v. 5.01 (SYSTAT Software Inc., Chicago, IL, USA). The determination coefficient (*R*^2^), the reduced chi-squared statistic (*χ*^2^), the root mean square error (*RMSE*), and the coefficient of residual variation (*CRV*) were used to evaluate the goodness of fit of the model:

(10) $$R^{2}=\frac{\sum_{i=1}^{N}{\left(MR_{i,p}-MR_{p}\right)}^{2}}{\sum_{i=1}^{N}{\left(MR_{i,e}-MR_{p}\right)}^{2}}$$

(11) $$\chi^{2}=\frac{\sum_{i=1}^{N}{\left(MR_{i,p}-MR_{i,e}\right)}^{2}}{N-n}$$

(12) $$RMSE=\sqrt{\frac{\sum_{i=1}^{N}{\left(MR_{i,p}-MR_{i,e}\right)}^{2}}{N}}$$

(13) $$CRV=100\%*\frac{\sqrt{\chi^{2}}}{Y}$$

The high *R*^2^ values and the lower *χ*^2^ and *RMSE* indicate that the model fits well with the experimental data. The values of *CRV* of less than 20% indicate that the model can be used for predictions.

### 2.4. Water Activity

Water activity was measured using an AquaLab CX-2 (Decagon Devices Inc., Pullman, DC, USA) apparatus, in accordance with the manufacturer’s instruction. The temperature of water activity determination was constant: 25 °C. Each measurement was conducted in four repetitions.

### 2.5. Statistical Analysis

The statistical software Statgraphics Plus ver. 5.1 (StatPoint, Warrenton, WV, USA) and Excel 2016 (Microsoft, Redmond, DC, USA) were used for data analysis. Pearson’s correlation coefficient between water activity and water content was calculated. The influence of pre-treatment (duration of the process, type of osmotic solution) on dependent variables: mass transfer parameters (*WC*, *WL*, *WL_T_*, *SG*, CR), as well as water activity, were evaluated by means of a multifactorial analysis of variance (ANOVA) at a significance level α = 0.05. In the case of significant impact factor, post-hoc Tukey’s test was performed.

## 3. Results and Discussion

### 3.1. Water Content

**Figure 1:**
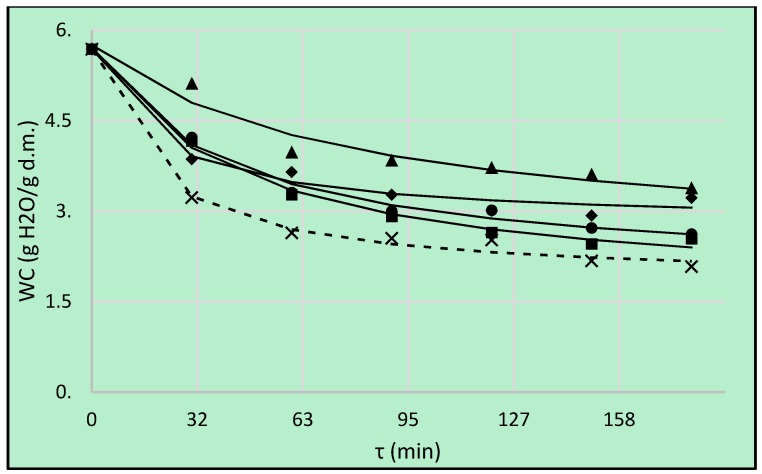
Water content (*WC*), kinetics using different solutions: erythritol (■), xylitol (●), maltitol (▲), dihydroxyacetone (◆), sucrose (**×**). Lines are the Peleg’s model. Dotted line is kinetic reference of sucrose.

The raw material of apples v. Elise was characterized by a *WC* of 5.69 ± 0.25 g H_2_O/g d.m. (Figure 1). This value was smaller than in the previous research by Cichowska et al. [10], using apples v. Paulared and in apples v. Champion [28]. The water content in the apple tissue decreased with prolonged time, however, higher changes were observed in the first 90 min of the process due to a high osmotic driving force between the fruit and the hypertonic surrounding medium. Water diffusivity may decrease at the beginning of the osmotic dehydration process due to the incorporation of sugar, but increase when the osmotic dehydration is carried out for more than one hour due to the breakdown of cells, which lowers the resistance to water diffusion [29]. Hypertonic solutions of erythritol (122.12 g/mol) and xylitol (152.15 g/mol) showed similar activity in decreasing of WC (Table 1). The highest degree of dehydration was achieved using these solutes—*WC* was reduced to about 2.5 g H_2_O/g d.m. It was slightly lower efficiency compared to sucrose solution. Additionally, concentration of polyols (30%) was much lower than sucrose solution (50%), which was related to lower molecular weight. Osmotic pressure depends on molar mass of the solute; the smaller the mass, the higher the pressure at the same concentration will be [30]. Moreover, xylitol has a lower viscosity in solution than sucrose at any given temperature or concentration [15]. Assis et al. [1] also observed the fact that the lower viscosity of the osmotic solution facilitates the mass transfer in the OD process.

In the previous research, the influence of concentration hypertonic solutions prepared using polyols group on the kinetics of OD was investigated [10]. In all cases, an increase in the osmotic solution concentration resulted in a greater degree of dehydration of the apples v. Paulared. When comparing values obtained during the first 3 h of the OD process in 30% concentrated osmotic agents, similar values for erythritol and sucrose (50%) solutions were observed. In the case of xylitol and maltitol, used as osmotic agents, *WC* in apple tissue were slightly smaller, which means that the reduction of water was higher. It could be explained that variety of the fruit can affect mass transfer during OD. However, Fanta et al. [31] reported that despite macroscopically observed variation in water conductivity, which was linked to variability in the microstructure of the tissue—differences in porosity, connectivity and cell distribution affected the water transport in the tissue to a minor extent. It also had a minor effect on the average water potential and water content.

Rodriguez et al. [32] osmo-dehydrated nectarines for 2 h (with an initial *WC* of 4.602 g/g d.m.) in a 40% concentrated sorbitol and glucose solutions. They achieved a reduction of *WC* in the tissue to the value of 2.59 g/g d.m. and 2.52 g/g d.m., respectively. These results were similar to the values observed in the present study, after 2 h duration of OD in 30% concentrated erythritol and xylitol, as well as 50% concentrated sucrose solutions (Figure 1).

Estimation of *WC* using Peleg’s model was effective in all cases—the goodness of fit was confirmed by high R^2^ values and low RMSE and χ^2^ values (Table 2). In addition, the values for parameters CRV were lower than 20%, which indicates that the model could be used for the prediction of *WC*. The Peleg constant k_1_ relates to the initial rate of dehydration. The Peleg constant k^2^ relates to the minimum attainable moisture content. In the studies carried out using Peleg’s model, the 1/k_1_ value well described the initial dehydration rate [22]. Dehydration rate at the very beginning of the process was high in the case of use sucrose and DHA, which was confirmed by the low values of parameter k_1_ amounting to 4.498 and 6.591, respectively (Table 2). More than two times greater value of k_1_ parameter characterized the modeling for maltitol as osmotic solution, compared to the most effective processes in erythritol and xylitol solutions. The values of k_2_ denote the equilibrium water content—the lower k_2_ parameter, the higher the water removal [10]. The lowest value of k_2_ was noted during OD in erythritol solution.

### 3.2. Water Loss

**Figure 2:**
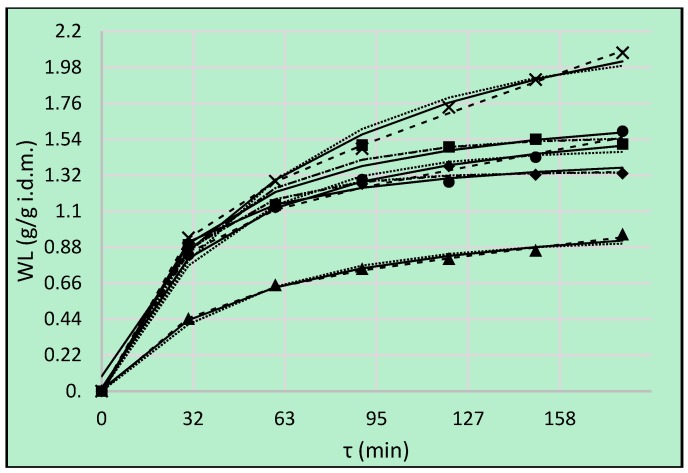
Water loss (*WL*), kinetics using different solutions: erythritol (■), xylitol (●), maltitol (▲), dihydroxyacetone (◆), sucrose (**×**). Lines are the Peleg’s model (solid lines), the Kelvin–Voigt model (dotted lines) and Burgers Model (dashed lines).

Water loss (*WL*) is the main parameter which indicates the amount of water removed during the osmotic pre-treatment in relation to the initial weight of the samples [29]. During OD the phenomenon of water loss mainly occurred. The highest value of *WL* above 2 g/g i.d.m. was obtained in the case of the reference process in 50% concentrated sucrose solution (Figure 2—**×** symbol). However, among non-conventional solutes used as osmotic agents, erythritol was the most effective in terms of *WL*. Compared with the group of polyols used in this research, erythritol had the lowest molecular weight—which gives it different properties, such as higher osmotic pressure and lower water activity in solution [12]. There were no significant differences between the use of xylitol and erythritol as hypertonic solutions, taking into account the final values of *WL* (Table 3). Time had significant influence on achieved values, mainly during the first 2 h. Cichowska et al. [10] also reported that osmotic pre-treatment for periods longer than 3 h was not effective. Pumpkin during OD needed approximately 150 min to reach the equilibrium values of water loss (*WL*) and solid gain (*SG*) [6]. Assis et al. [1], who studied the OD of apple cubes in sucrose and sorbitol solutions, reported a rapid increase in *WL* and *SG* at the beginning of the process. They also observed that the initial rate of *WL* was higher when sorbitol was used as the osmotic agent (60% concentrated solution), compared to sucrose in the same conditions. This phenomenon was explained by the lower viscosity of sorbitol solution at the beginning of the process. In addition, the molar concentration of the sorbitol solution (3.294 M) was higher than the one of sucrose solution (1.753 M) for the same mass concentration.

In the previous research Cichowska et al. [10] osmo-dehydrated apples of another variety and then they achieved higher values of *WL*. For example, after three hours of the OD process the highest value above 3 g/g i.d.m. was found for sucrose solution used as the reference. The values of *WL* reported in the case of erythritol and xylitol solutions in 20% concentrate were similar to those observed for the same solutes in 30% concentrate in the present experiment. Moreover, 40% and 30% concentrated erythritol and xylitol, as well as 40% concentrated maltitol solutions were more effective, compared to the best kinetics for water remove (Figure 2—sucrose) in present study. Despite this, the kinetics of 40% concentrated inulin solution, which was considered to be ineffective in the previous research, was still more effective compared to the kinetics of *WL* in the case of maltitol in the present study. It could be explained by the fact that the concentration of maltitol was lower than the concentration of sucrose at similar molecular weight of both compounds amounting to 34,431 g/mol and 34,230 g/mol, respectively. However, the use of higher concentration of maltitol, for example 60%, gave the possibility to achieve even higher values of *WL*, compared to sucrose in the same concentration [9]. On the other hand, after OD of apples cv. Champion for 90 min at 45 °C in 40% concentrated solutions of sucrose and chokeberry juice—achieved values of *WL* were over 4 times smaller [28], compared to present results. This means that *WL* kinetics depends not only on processing parameters, but also on the fruit variety.

To evaluate the goodness of fit of the experimental points of OD in different solutions three models were analyzed. The Peleg’s model is commonly used by researchers to osmotic dehydration process modeling [1,9,10]. The Kelvin–Voigt model is a basic fractional model, which is used in rheology. The Kelvin–Voigt model is the simplest viscoelastic body of type III. This model exhibits an exponential (reversible) strain creep but no stress relaxation; it is also referred to as the retardation element [27]. The expanded Kelvin–Voigt model, also used in rheology, is a Burgers model which is obtained by adding a dashpot or a spring to the representations of the Zener or of the anti-Zener model, respectively [27]. These models could be used in OD process modeling, because of the goodness of fit of experimental data (Table 4) and physical meaning of their parameters. Namely, parameter A represents the equilibrium and retardation value in Kelvin–Voigt and Burgers model, respectively. Parameter B is a time constant, which indicates the time necessary to obtain 37% of equilibrium or retardation value achieved at a decreasing rate period. The lower the B value the higher the rate of change at the beginning of the process. Parameter C is the velocity of change at the constant rate period, which can be described only by the Burgers model. CRV values lower than 20% indicate the usefulness of all tested models for the prediction of water loss (Table 4). However, the lowest CRV values as well as RMSE were found for Burgers model. This particularly regards OD in xylitol, maltitol and sucrose solutions. In those cases equilibrium was not achieved within 180 min of experiment and the highest increase of *WL* at the constant rate period (Figure 2) confirmed by the highest value of C (Table 4) was stated for sucrose solution. Considering that *1*/*k*_1_ in a Peleg’s model, which is the most recognized prediction tool for OD characteristic, describes the initial mass transfer rate, the *WL* rate using DHA and erythritol solutions were higher at the beginning of the process. However, the calculated values for *k*_1_ parameter were much higher, compared to those reported by Assis et al. [1], where *k*_1_-values were in the ranges of 0.970–4.560. The value of *1*/*k*_2_, which is related to the equilibrium value, was the highest for apples dehydrated in sucrose solution, compared to other solutes used.

Dermesonlouoglou and Giannakourou used multi-component aqueous solutions, containing glycerol, erythritol, sodium chloride, calcium chloride, steviol glucoside, and Citrox during the process of osmotic dehydration of apricot [33] and peach [34]. They observed higher values of *WL* just after 20 min of the OD in every concentration of the hypertonic solution (40–60%) and at every temperature (25–45 °C), compared to present results. This phenomenon was reported in the case of both apricot and peach.

### 3.3. Solid Gain

**Figure 3:**
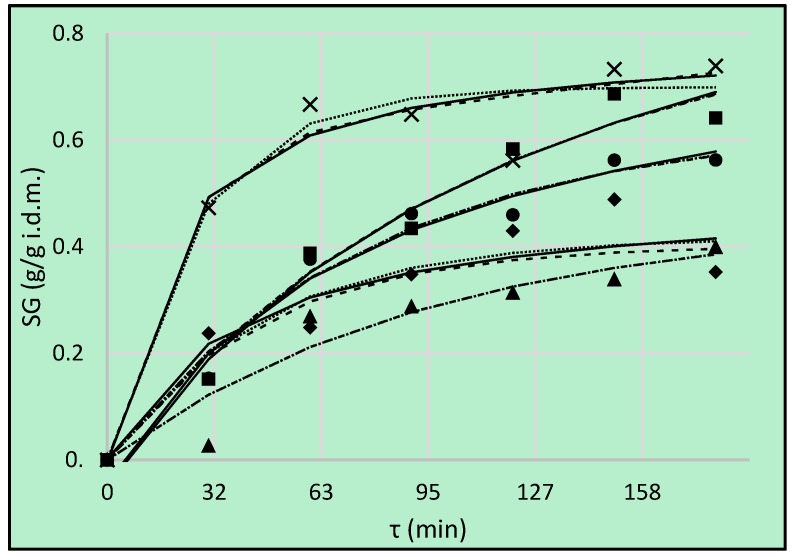
Solid gain (*SG*), kinetics using different solutions: erythritol (■), xylitol (●), maltitol (▲), dihydroxyacetone (◆), sucrose (**×**). Lines are the Peleg’s model (solid lines), the Kelvin–Voigt model (dotted lines) and Burgers Model (dashed lines).

Sugar gain is an important parameter that indicates the amount of soluble solids that are incorporated by the sample during the pre-treatment. Generally, the performance of the pre-treatment will be satisfactory when high water loss is associated with low sugar gain [29]. The values of *SG* were lower, compared to parameter *WL*, discussed above. It is worth mentioning that the phenomenon of water loss was observed mainly during the first 2 h, but in the case of *SG*, solid uptake in the third hour was still noticed (Figure 3). The highest values were achieved, when sucrose solution was used as an osmotic agent. Nevertheless, the main aim of the present research was to remove some water from the apple tissue, but not to enrich in sugar compounds. The use of erythritol and xylitol solutions gave similar results from the statistical point of view—the values were classified into one homogenous group (Table 5).

Reported in present research, the values of *SG* for reference kinetic in sucrose solution were close to those obtained in the previous experiment of the Authors [10]. However, the values noted in the present research in the case of erythritol, xylitol and maltitol were lower, compared to OD in the same conditions (concentrate, temperature and type of osmotic agent) in the previous study. Actual kinetics courses of *SG* were similar to those reported by Dermesonlouoglou and Giannakourou [33] during OD of apricot pieces at 25 °C. Similarity could be seen between OD in 60, 50, and 40% concentrated solution (mixture containing mainly glycerol with erythritol) and OD in the cases of erythritol, xylitol, DHA in present study, respectively. The phenomenon of solid gain was higher than in research by Lech et al. [28], where *SG* values after 90 min of OD of apples were over 7 times lower. On the other hand, the values of *SG* were lower, compared to the ones achieved by Katsoufi et al. [6], but they osmo-dehydrated pumpkin at much higher temperatures (75–95 °C).

Peleg’s model cannot be used for the prediction of OD process in the case of maltitol used as an osmotic agent, because CRV value was greater than 20% (Table 6). The highest initial rate of *SG* was in the case of sucrose solution (the lowest values of *k*_1_ and B), which is showed also in Figure 3. Erythritol showed higher equilibrium solid content than sucrose, when taking into account values *1*/*k*_2_ and A. Similar phenomenon was also observed by Assis et al. [1] in the case of sorbitol and sucrose. It was explained by the fact that smaller molecules diffuse more easily than larger ones (the molecular weight of erythritol is almost three times smaller than MW of sucrose). On the other hand, the highest values of C observed for sucrose means that this substance clearly demonstrates a constant rate period and therefore has the highest penetrating potential for the longer duration of osmotic pre-treatment.

### 3.4. True Water Loss

**Figure 4:**
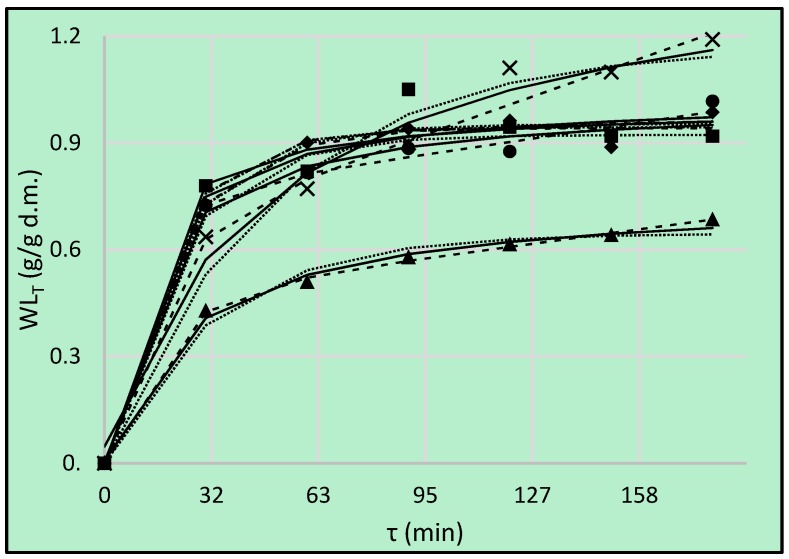
True water loss (*WL_T_*), kinetics using different solutions: erythritol (■), xylitol (●), maltitol (▲), dihydroxyacetone (◆), sucrose (**×**). Lines are the Peleg’s model (solid lines), the Kelvin–Voigt model (dotted lines) and Burgers Model (dashed lines).

During the process of the osmotic dehydration three simultaneous mass transfers occur: water transfers from the product to the solution, the solute transfers from the solution to the product, and the product’s own solutes (sugars, organic acids, minerals, vitamins, etc.) leach out. However, this third process is negligible compared with the first two transfers [35]. Sometimes the main reason for the use OD as pre-treatment is an enrichment in valuable compounds, among others probiotics and bioactive molecules [36,37]. On the other hand, for some researchers it was more important to remove some water from the tissue than solid gain [10]. Therefore, it was also in the present case. The proposed parameter of True Water Loss includes actual solid gain during the process and it was calculated according to Equation (4). The values of this parameter are shown in Figure 4. This parameter also presents the predominance of water loss over solids gain during OD. The similar activity showed all the used hypertonic solutions, except the solution contained maltitol. This was confirmed by statistical analysis—only values achieved during OD in maltitol were classified into another homogeneous group (Table 7). Based on statistical results, it could also be stated that the real water loss took place within 2 first hours—OD carried out longer than this period was unfounded.

Peleg’s model is suitable also for prediction of *WL_T_* parameter. However, the lowest values of CRV and RMSE were found for Burgers model (Table 8). The initial rate of dehydration was the highest in the case of erythritol (the lowest value of k_1_ and relatively low values of B), but sucrose showed higher equilibrium solid content (the highest value of 1/*k*_2_ and relatively low value of B for Kelvin–Voigt model). This is an opposite situation compared to parameter *SG* discussed above, resulted from including solid uptake to an actual value of water loss—which also caused lower values of *WL_T_* (Figure 4) compared to *WL* (Figure 2).

### 3.5. Cichowska’s et al. Ratio

**Figure 5:**
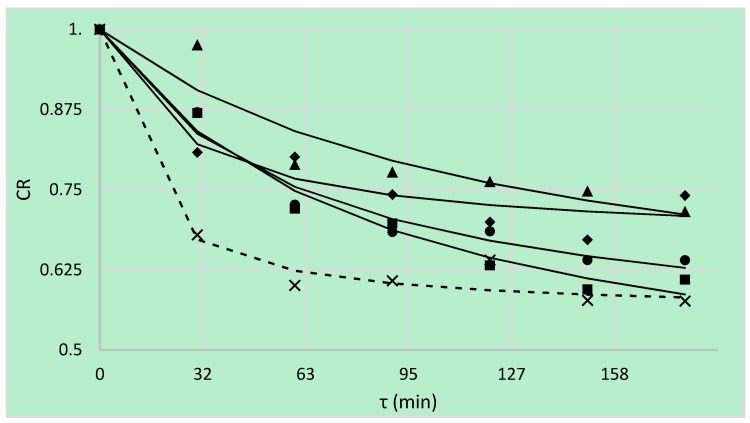
Cichowska’s et al. ratio (CR), kinetics using different solutions: erythritol (■), xylitol (●), maltitol (▲), dihydroxyacetone (◆), sucrose (**×**). Lines are the Peleg’s model. Dotted line is kinetic reference of sucrose.

Proposed Cichowska’s et al. ratio (CR) defined as the ratio of true water loss over water loss taking into account solid gain during the process. In the case of the highest enrichment in OD solutes, CR value should be as small as possible (because of the lower value of a parameter of true water loss). However, in the case of this research, there should be the opposite situation. Since the aim was not enrichment with sugar alcohols, the ratio should be possibly highest. It is also important that alone value of the CR is not sufficient. The interpretation of this parameter requires taking into account the water loss and solid gain as well. For example, it can be seen that most values of CR were obtained in the case of OD in maltitol solution (Figure 5). Nevertheless, maltitol was not the most effective osmotic agent. Indeed, the solid gain in this case was marginal, but the water loss was lower than expected. It did not affect significantly the value of true water loss and, consequently, the CR. Although the values of the true water loss (Figure 4) for erythritol, xylitol and DHA were similar—the solution of dihydroxyacetone showed the best behavior (the coefficient was higher, compared to listed solutions).

The model presented by Peleg can also be used to model the values of the new parameter Cichowska’s et al. ratio. The values of *k*_1_ parameter were different (Table 9). If water removal was the main aim the parameter *k*_1_ and *k*_2_ should be as high as possible. Maltitol and DHA used as osmotic agents fulfilled most of these expectations. However, DHA exhibited the higher value of *WL* and thus can be recommended as the best osmotic solution because possibly high *WL* at relatively low *SG* were achieved during OD process.

Statistical analysis showed a significant influence of osmotic agent as well as time on achieved values of CR (Table 10). In the case of sucrose solution, obtained values were the lowest, because of high solid gain during OD. Statistically significant differences between use of erythritol and xylitol were not observed. Tuckey’s test also confirmed that process lasting longer than 2 h was not effective.

### 3.6. Water Activity

**Figure 6:**
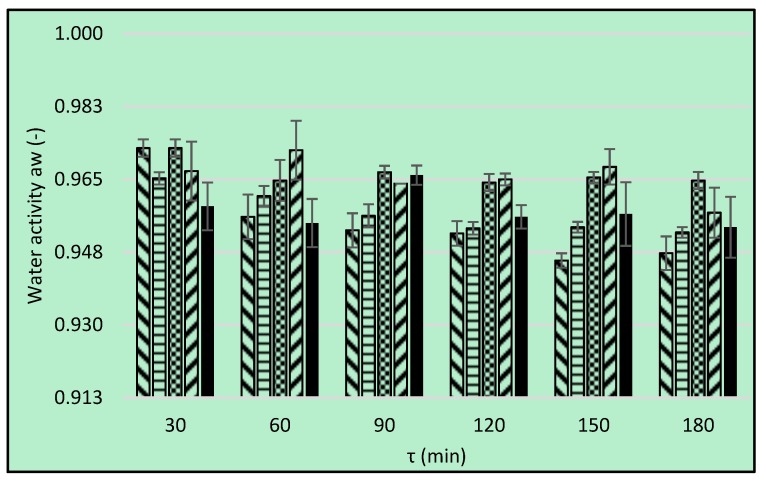
Water activity, a_w_, bars present the use of different solutions: erythritol (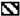), xylitol (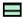), maltitol (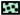), dihydroxyacetone (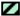), sucrose (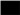). Dotted line is an initial a_w_ of raw apple tissue.

The raw material used in the present study was characterized by the water activity of 0.967 (Figure 6—dotted line). The value of this parameter is not the quantity of water in food but its thermodynamic state that is responsible for its influence on food stability and texture. The osmotic dehydration does not reduce water activity sufficiently to hinder the proliferation of microorganisms. The process extends, to some degree, the shelf life of the material, but it does not preserve it [30]. Increasing the time of OD process resulted in clear decrease of water activity values in the case of erythritol and xylitol (Figure 6). For other osmotic agents the time influence was ambiguous. Statistically, 120 min of the process was found as the most effective time (Table 11). Higher values of a_w_ in the case of maltitol was also observed by Cichowska et al. [10] and it was related to small *WL* during OD. Generally, the values obtained in the previous research were lower, compared to the present results achieved in similar conditions. Statistical significant differences between the values of aw obtained during OD process in sucrose, erythritol and xylitol were not found (Table 11). However, the values noticed when polyols (erythritol and xylitol) as osmotic agents were used, were lower than in the case of sucrose solution. The results are in agreement with those obtained by Assis et al. [1]—a_w_ of osmotically dehydrated in sorbitol solutions samples were lower than in sucrose solutions. The *WL* and *SG* observed during osmotic dehydration caused a decrease in the a_w_ of the fruit also in the case of bananas dehydrated in sucrose solution for 10–300 min [36]. Katsoufi et al. [6] also stated that the water activity of osmotically dehydrated pumpkin was found to decrease during processing. Osmotic pre-treatment substantially lowered water activity with the increase of time, temperature and glycerol concentration in the case of osmo-dehydrated apricot and peach tissue [33,34] and the achieved values were much lower, compared to present results. The values of a_w_ observed by Lech et al. [28] after 90 min of OD of apples in 40% concentrated solutions amounted to 0.971 and 0.959 for sucrose and chokeberry juice, respectively.

Pearson’s correlation coefficient between water activity and water content was calculated separately for each type of osmotic solution. In the case of erythritol, xylitol, and maltitol, the linear relationship between the variables was observed. The strongest relationship was observed during OD in erythritol solution (correlation coefficient 0.909) In the case of xylitol and maltitol, the correlation coefficients were equal 0.866 and 0.695, respectively. Pearson’s correlation between a_w_ and *WC* in the case of OD in sucrose (*p*-Value = 0.1943) and DHA (*p*-Value = 0.5616) solutions was not observed. In another research, the authors also observed these relationships, but the Pearson’s coefficients were weaker [10]. Assis et al. [1] stated that the a_w_ of the dehydrated samples decreased when their final water content decreased, and the determination coefficients of the linear correlation were between 0.656 and 0.982. The lack of a clear correlation between water activity and water content for some cases results from the fact that the assays were conducted in the range of relatively high water content with the ambiguous effect of the type of osmotic substances.

## 4. Conclusions

Solutions of erythritol and xylitol in 30% concentrate could be an alternative to sucrose in the process of osmotic dehydration. Their activity as osmotic agent were satisfactory, including water loss, small solid uptake (compared to sucrose) as well as decreasing values of water activity. In the tested concentration, maltitol solution was ineffective, but DHA showed the ability to remove some water from the apple tissue at the relatively low solid gain. Therefore, DHA can be recommended as osmotic agent for the OD process, whose first aim is water removal without chemical alterations, resulting from penetration of solid particles into the fruit tissue. The advantages of using osmotic agents mentioned above are: higher water loss values compared to maltitol solution as well as effect on fruit characteristics such as sensory profile. True water loss, compared to water loss, can describe better and be helpful to analyze the phenomenon of the mass exchange during the OD process. In addition, Cichowska’s et al. Ratio could be used to evaluate examined solutions in terms of different aims of the OD considering water removal and incorporation of solids from osmotic solution. Modeling of mass transfer parameters, using Peleg’s model can be satisfactorily supplemented by Kelvin–Voigt and Burgers model for better prediction of OD within the particular periods of the process. Particularly, Burgers model can describe OD process at the constant rate period, which cannot be distinguished using other models.

## Figures and Tables

**Table 1:** The influence of osmotic agents and pre-treatment time on water content in fruit.

Factor		*p*-Value	Contrast	+/− Limits	Difference
Type of osmotic substance	Erythritol ^b^	0.000 *	erythritol-xylitol	0.1506	−0.1494
	Xylitol ^b^		erythritol-sucrose	0.1563	0.4871 *
	Maltitol ^d^		xylitol-maltitol	0.1506	−0.7871 *
	DHA ^c^		xylitol-sucrose	0.1563	0.6365 *
	Sucrose ^a^		DHA-erythritol	0.1798	0.3516 *
Time (min)	30 ^d^	0.000 *	30–60	0.1818	0.8063 *
	60 ^c^		60–90	0.1818	0.2404 *
	90 ^b^		90–120	0.1879	0.1443
	120 ^b^		120–150	0.1901	0.1965 *
	150 ^a^		120–180	0.1856	0.2175 *
	180 ^a^		150–180	0.1823	0.0210

Statistical differences between factors; a Tukey test of main effects was performed. * denotes a statistically significant difference. Means within columns with a different lowercase letter superscript are significantly different (*p* < 0.05).

**Table 2:** The values of *k_1_*, *k_2_, R^2^*, *χ^2^,* coefficient of residual variation (CRV) and root mean square error (RMSE) of modeling Water content (*WC*) using Peleg’s model. DHA: dihydroxyacetone.

Solution	Model	*k*_1_ (kg/kg)	*k*_2_ (kg/kg·h)	*R* ^2^	*χ* ^2^	CRV (%)	RMSE
Erythritol	Peleg	10.892	0.242	0.981	0.009	2.88	0.082
Xylitol	Peleg	10.804	0.264	0.970	0.013	3.20	0.095
Maltitol	Peleg	22.588	0.294	0.933	0.041	4.84	0.171
DHA	Peleg	6.591	0.344	0.970	0.024	3.97	0.130
Sucrose	Peleg	4.498	0.260	0.982	0.013	3.77	0.097

**Table 3:** The influence of osmotic agents and pre-treatment time on water loss during osmotic dehydration (OD).

Factor		*p*-Value	Contrast	+/− Limits	Difference
Type of osmotic substance	Erythritol ^c^	0.000 *	erythritol-xylitol	0.1054	0.0887
	Xylitol ^bc^		erythritol-sucrose	0.1094	−0.2086 *
	Maltitol ^a^		xylitol-maltitol	0.1054	0.5168 *
	DHA ^b^		xylitol-sucrose	0.1094	−0.2973 *
	Sucrose ^d^		DHA-erythritol	0.1259	−0.1397 *
Time (min)	30 ^a^	0.000 *	30–60	0.1273	−0.2666 *
	60 ^b^		60–90	0.1273	−0.2010 *
	90 ^c^		90–120	0.1315	−0.0672
	120 ^cd^		120–150	0.1331	−0.0871
	150 ^de^		120–180	0.1299	−0.1758 *
	180 ^e^		150–180	0.1276	−0.0887

Statistical differences between factors; a Tukey test of main effects was performed. * denotes a statistically significant difference. Means within columns with a different lowercase letter superscript are significantly different (*p* < 0.05).

**Table 4:** The values of *k*_1_, *k*_2_*, R*^2^, *χ*^2^, CRV and RMSE of modeling *WL* using Peleg’s, Kelvin–Voigt and Burgers model.

Solution	Model	*k*_1_ (kg/kg)	*k*_2_ (kg/kg·h)	A	B	C (10^−3^)	*R* ^2^	*χ* ^2^	CRV (%)	RMSE
Erythritol	Peleg	16.714	0.534	-	-	-	0.862	0.006	5.85	0.064
	Kelvin–Voigt	-	-	1.557	37.648	-	0.870	0.004	5.07	0.055
	Burgers	-	-	1.555	37.460	0.000	0.870	0.004	5.08	0.055
Xylitol	Peleg	21.117	0.558	-	-	-	0.942	0.004	5.26	0.054
	Kelvin–Voigt	-	-	1.482	41.120	-	0.920	0.007	7.07	0.072
	Burgers	-	-	0.957	19.818	3.304	0.955	0.002	3.72	0.038
Maltitol	Peleg	45.191	0.846	-	-	-	0.910	0.001	3.18	0.019
	Kelvin–Voigt	-	-	0.932	51.859	-	0.899	0.001	4.87	0.029
	Burgers	-	-	0.574	26.915	2.038	0.915	0.000	1.94	0.012
DHA	Peleg	13.019	0.658	-	-	-	0.976	0.002	3.51	0.034
	Kelvin–Voigt	-	-	1.340	28.683	-	0.978	0.001	3.26	0.031
	Burgers	-	-	1.319	27.946	0.132	0.978	0.001	3.25	0.031
Sucrose	Peleg	28.202	0.362	-	-	-	0.974	0.005	4.88	0.060
	Kelvin–Voigt	-	-	2.113	63.358	-	0.958	0.009	6.39	0.079
	Burgers	-	-	0.941	19.074	6,332	0.987	0.000	1.40	0.017

**Table 5:** The influence of osmotic agents and pre-treatment time on solid gain during OD.

Factor		*p*-Value	Contrast	+/− Limits	Difference
Type of osmotic substance	Erythritol ^c^	0.000 *	erythritol-xylitol	0.0578	0.0511
	Xylitol ^c^		erythritol-sucrose	0.0600	−0.1692 *
	Maltitol ^a^		xylitol-maltitol	0.0578	0.1574 *
	DHA ^b^		xylitol-sucrose	0.0600	−0.2203 *
	Sucrose ^d^		DHA-erythritol	0.0690	−0.1381 *
Time (min)	30 ^a^	0.000 *	30–60	0.0698	−0.2003 *
	60 ^b^		60–90	0.0698	−0.0405
	90 ^bc^		90–120	0.0721	−0.0428
	120 ^cd^		120–150	0.0730	−0.0837 *
	150 ^e^		120–180	0.0712	−0.0655
	180 ^de^		150–180	0.0700	0.0182

Statistical differences between factors; a Tukey test of main effects was performed. * denotes a statistically significant difference. Means within columns with a different lowercase letter superscript are significantly different (*p* < 0.05).

**Table 6:** The values of *k*_1_, *k*_2_, *R*^2^, χ^2^, CRV and RMSE of modeling solids gain (*SG*), using Peleg’s, Kelvin–Voigt and Burgers model.

Solution	Model	*k*_1_ (kg/kg)	*k*_2_ (kg/kg·h)	A	B	C (10^−3^)	*R* ^2^	*χ* ^2^	CRV (%)	RMSE
Erythritol	Peleg	103.136	0.782	-	-	-	0.906	0.002	10.65	0.041
	Kelvin–Voigt	-	-	0.871	115.812	-	0.906	0.002	9.81	0.038
	Burgers	-	-	0.863	114.045	0.000	0.906	0.002	9.81	0.038
Xylitol	Peleg	89.879	1.108	-	-	-	0.899	0.002	9.88	0.034
	Kelvin–Voigt	-	-	0.634	77.564	-	0.901	0.001	8.75	0.030
	Burgers	-	-	0.632	77.055	0.000	0.901	0.001	8.75	0.030
Maltitol	Peleg	138.805	1.398	-	-	-	0.849	0.004	23.47	0.052
	Kelvin–Voigt	-	-	0.456	96.233	-	0.843	0.003	19.81	0.044
	Burgers		-	0.455	95.800	0.000	0.843	0.003	19.82	0.044
DHA	Peleg	79.275	1.975	-	-	-	0.757	0.003	18.20	0.050
	Kelvin–Voigt	-	-	0.418	45.490	-	0.763	0.003	17.98%	0.049
	Burgers	-	-	0.399	44.420	0.020	0.755	0.004	18.84%	0.052
Sucrose	Peleg	22.821	1.254	-	-	-	0.802	0.004	10.63%	0.055
	Kelvin–Voigt	-	-	0.699	25.840	-	0.799	0.004	10.92%	0.056
	Burgers	-	-	0.599	19.488	0.704	0.811	0.004	10.03%	0.052

**Table 7:** The influence of osmotic agents and pre-treatment time on true water loss during OD.

Factor		*p*-Value	Contrast	+/− Limits	Difference
Type of osmotic substance	Erythritol ^b^	0.000 *	erythritol-xylitol	0.0751	0.0332
	Xylitol ^b^		erythritol-sucrose	0.0779	−0.0306
	Maltitol ^a^		xylitol-maltitol	0.0751	0.2969 *
	DHA ^b^		xylitol-sucrose	0.0779	−0.0639
	Sucrose ^b^		DHA-erythritol	0.0896	−0.0080
Time (min)	30 ^a^	0.000 *	30–60	0.0907	−0.0969 *
	60 ^b^		60–90	0.0907	−0.1146 *
	90 ^c^		90–120	0.0937	−0.0183
	120 ^cd^		120–150	0.0948	−0.0059
	150 ^cd^		120–180	0.0925	−0.0735
	180 ^d^		150–180	0.0909	−0.0676

Statistical differences between factors; a Tukey test of main effects was performed. * denotes a statistically significant difference. Means within columns with a different lowercase letter superscript are significantly different (*p* < 0.05).

**Table 8:** The values of *k*_1_, *k*_2_, *R*^2^, *χ*^2^, CRV and RMSE of modeling *WL_T_* using Peleg’s, Kelvin–Voigt and Burgers model.

Solution	Model	*k*_1_ (kg/kg)	*k*_2_ (kg/kg·h)	A	B	C (10^−3^)	*R* ^2^	*χ* ^2^	CRV (%)	RMSE
Erythritol	Peleg	8.734	0.993	-	-	-	0.723	0.005	7.94	0.058
	Kelvin–Voigt	-	-	0.944	18.425	-	0.728	0.004	7.67	0.056
	Burgers	-	-	0.942	18.163	0.000	0.728	0.004	7.67	0.056
Xylitol	Peleg	13.276	0.982	-	-	-	0.899	0.001	4.62	0.033
	Kelvin–Voigt	-	-	0.923	21.465	-	0.870	0.003	6.49	0.046
	Burgers	-	-	0.733	11.139	1.417	0.917	0.001	2.99	0.021
Maltitol	Peleg	34.796	1.338	-	-	-	0.860	0.000	3.23	0.015
	Kelvin–Voigt	-	-	0.646	32.687	-	0.833	0.001	5.94	0.028
	Burgers	-	-	0.455	15.824	1.276	0.868	0.000	1.51	0.007
DHA	Peleg	10.983	0.966	-	-	-	0.958	0.002	4.67	0.033
	Kelvin–Voigt	-	-	0.953	20.798	-	0.962	0.001	3.87	0.028
	Burgers	-	-	0.923	19.484	0.209	0.963	0.001	3.87	0.028
Sucrose	Peleg	36.246	0.696	-	-	-	0.959	0.003	6.50	0.049
	Kelvin–Voigt	-	-	1.174	49.883	-	0.934	0.005	7.93	0.059
	Burgers	-	-	0.608	14.691	3.340	0.974	0.002	5.48	0.041

**Table 9:** The values of *k*_1_, *k*_2_, *R*^2^, *χ*^2^, CRV and RMSE of modeling Cichowska’s et al. ratio (CR) using Peleg’s model.

Solution	Model	*k*_1_ (kg/kg)	*k*_2_ (kg/kg·h)	*R* ^2^	*χ* ^2^	CRV (%)	RMSE
Erythritol	Peleg	139.433	1.645	0.959	0.000	3.28	0.019
Xylitol	Peleg	124.570	1.997	0.942	0.000	3.23	0.020
Maltitol	Peleg	255.096	2.046	0.877	0.002	5.05	0.034
DHA	Peleg	77.326	3.007	0.893	0.001	4.15	0.027
Sucrose	Peleg	23.758	2.260	0.967	0.001	3.83	0.020

**Table 10:** The influence of osmotic agents and pre-treatment time on CR during OD.

Factor		*p*-Value	Contrast	+/− Limits	Difference
Type of osmotic substance	Erythritol ^b^	0.000 *	erythritol-xylitol	0.0309	-0.0205
	Xylitol ^b^		erythritol-sucrose	0.0320	0.0804 *
	Maltitol ^d^		xylitol-maltitol	0.0309	−0.0863 *
	DHA ^c^		xylitol-sucrose	0.0320	0.1008 *
	Sucrose ^a^		DHA-erythritol	0.0369	0.0605 *
Time (min)	30 ^d^	0.000 *	30–60	0.0373	0.1249 *
	60 ^c^		60–90	0.0373	0.0221
	90 ^bc^		90–120	0.0385	0.0222
	120 ^ab^		120–150	0.0390	0.0336
	150 ^a^		120–180	0.0380	0.0254
	180 ^a^		150–180	0.0374	−0.0082

Statistical differences between factors; a Tukey test of main effects was performed. * denotes a statistically significant difference. Means within columns with a different lowercase letter superscript are significantly different (*p* < 0.05).

**Table 11:** The influence of osmotic agents and pre-treatment time on water activity during OD.

Factor		*p*-Value	Contrast	+/− Limits	Difference
Type of osmotic substance	Erythritol ^a^	0.000 *	erythritol-xylitol	0.0042	−0.0026
	Xylitol ^a^		erythritol-sucrose	0.0044	−0.0030
	Maltitol ^b^		xylitol-maltitol	0.0042	−0.0095 *
	DHA ^b^		xylitol-sucrose	0.0044	−0.0004
	Sucrose ^a^		DHA-erythritol	0.0050	0.0109 *
Time (min)	30 ^c^	0.000 *	30–60	0.0051	0.0067 *
	60 ^b^		60–90	0.0051	−0.0003
	90 ^b^		90–120	0.0052	0.0036
	120 ^ab^		120–150	0.0053	0.0007
	150 ^ab^		120–180	0.0052	0.0027
	180 ^a^		150–180	0.0051	0.0020

Statistical differences between factors; a Tukey test of main effects was performed. * denotes a statistically significant difference. Means within columns with a different lowercase letter superscript are significantly different (*p* < 0.05).

## Nomenclature

OD	osmotic dehydration,
*WC*	water content, (g H_2_O/g d.m.)
*WL*	water loss, (g/g i.d.m.)
*SG*	solids gain, (g/g i.d.m.)
CR	Cichowska’s et al. Ratio
*m*	sample mass, (g)
*s*	dry solids mass of material, (g)
*τ*	time of osmotic dehydration, (min)
*k*_1_	parameter of Equation (6), (kg/kg)
*k*_2_	parameter of Equation (6), (kg/kg*h)
MR_i,p_	predicted value of *WC*, *WL*, *SG*, *WL_T_* or CR
MR_i,e_	experimental value of *WC*, *WL*, *SG*, *WL_T_* or CR
N	number of observation
n	number of constants in the model equation
A, B, C	parameters in Kelvin–Voigt and Burgers models
Y	mean experimental value of *WC*, *WL*, *SG*, *WL_T_* or CR, respectively (in Equations (6)–(9))
**Subscripts**	
o	initial
τ	time (min)
T	true
